# The Effect of Health Promoting Programs on Patient’s Life Style After Coronary Artery Bypass Graft–Hospitalized in Shiraz Hospitals

**DOI:** 10.5539/gjhs.v8n5p154

**Published:** 2015-09-28

**Authors:** Leila Safabakhsh, Azizollah Arbabisarjou, Mozhgan Jahantigh, Mahshid Nazemzadeh, Shahindokht Navabi Rigi, Shahin Nosratzehi

**Affiliations:** 1 Health Promotion Research Center, Zahedan University of Medical Sciences of Zahedan, Zahedan, IR Iran; 2 Nursing & Midwifery School, Pregnancy Health Research Center, Zahedan University of Medical Sciences Zahedan, IR Iran; 3 Health Promotion Research Center, Zahedan University of Medical Sciences of Zahedan, Zahedan, IR Iran

**Keywords:** Coronary Artery Bypass Graft, HPP, lifestyle, education

## Abstract

**Background:**

Health promotion is an essential strategy for reduction of health disparities. Health promotion includes all activities that encourage optimum physical, spiritual, and mental functions. The aim of this study was to determine the impact of a Health Promotion Program (HPP) on behavior in terms of the dimensions of the Health Promoting Lifestyle Profile (HPLP) in patients after Coronary Artery Bypass Graft (CABG).

**Methods and Materials:**

In this clinical trial study, 80 patients who had undergone CABG surgery (2011-2012) were selected and randomly divided in two groups: Experimental and Control that investigated by (HPLP II). Then the experimental group was educated about diet, walking and stress management. The program process was followed up for three months and after tward whole variables were investigated again. The overall score and the scores for the six dimensions of the HPLP (self actualization, health responsibility, exercise, nutrition, interpersonal support and stress management) were measured in the pre- and post-test periods. Data were manually entered into SPSS version 21(IBM Corp, USA) by one the authors. Statistical analysis was performed using Student’s t-test and paired t-test. Mean standard deviation and standard error of the mean (with 95% Confidence Interval) were generated for each item.

**Results:**

Results showed that score of stress management (p=.036), diet (p=.002), Spiritual Growth (p=.001) and interrelationship (p=002) increase in experimental group after intervention. Average scores after three months in the control group had no significant changes; except responsibility for health (p<.05). Results of the study revealed that comparison the scores of the experimental group were significantly different from the control group in all lifestyle aspects except for spiritual growth.

**Conclusion:**

This study showed that HPP on lifestyle and health promotion in patients who suffered from Coronary Heart Disease (CAD) could improve the patient’s awareness of healthy behaviors and well-being in the quality of life.

## 1. Introduction

CAD is the leading cause of morbidity and mortality in the world (Ades, 2001). Increasing prevalence of CAD reflects the effects of the many risk factors that are becoming more common such as type 2 diabetes mellitus, obesity, sedentary lifestyle, stress and fatty diet (Mozaffarian, Wilson, & Kannel, 2008). Clinical evidences demonstrated that a sedentary lifestyle, stress and fatty diet are significant modifiable risk factor for CHD (Weberg, Hjermstad, Hilmarsen, & Oldervoll, 2013). Cardiac rehabilitation and HPPs are the most common form which they medically supervised exercise programs, stress management and diet that have been shown to improve health and decrease risk factors (Koch, Li, Lauer, Sabik, Starr, & Blackstone, 2007). By this way HPPs can lead to greater self-esteem, self-efficacy, and self-confidence and, then leading in anxiety reducing (Sol, van der Graaf, van Petersen, & Visseren, 2011). Health education facilitates health promotion and change the community lifestyle and high-risk behaviors for preventing disease. Some patients need to modify life style, for example, in relation to nutrition, exercise, alcohol and tobacco (Whitehead, 2007). Pishkarmofrad et al. (2012) in their study titled” CAD in critical pateints of Iran, found that there has been a significant relationship between hypertension (HTN), Hyperlipidemia, diabetes mellitus (DM), obesity and gender. Healthy lifestyle allows improvement of health and quality of life well-being at any stage of growth and development. The factors of health-promoting which have been considered in the intervention and evaluation of the results are: self - actualization, health responsibility, exercise, nutrition, interpersonal support and stress management (Carreno, Vyhmeister, Grau, & Ivanovic, 2006).

Oliver-McNeil & Artinian (2002) used the Health-Promoting Lifestyle Profile II (HPLP II; Walker & Pender) to study HPPs in women with CHD and reported the highest mean item scores in the subscale of stress management and interpersonal relations. Education and health promotion programs conducted by Brown have proved that participants in cardiac rehabilitation (experimental group) returned to work earlier than not participants (control group) (Brown, 2001). Impact of comprehensive lifestyle modification did not consider in clinical procedure and additional studies are required to clarify beneficial effect of lifestyle modification (Brown et al., 2004). To reduce the risk of developing more disease in the future, the advice to the patient certainly is to stop smoking, reduce consumption of fat and cholesterol. A survey has demonstrated that 90% of patients with past known CAD had at least one or two high risk lifestyles related CVD such as smoking, fatty diet consumption, immobility or a sedentary lifestyle (Yusuf et al., 1994).

Although numerous studies have investigated the outcome of CABG, but a little attention has been given to patient’s health promotion and lifestyle modification after CABG. While the pain relieved after CABG, all training and teaching programs have given by nurses to patients about wound dressing, wound healing, activities and exercises, taking medications, postoperative complications and cares. But it has been done less attention and consideration to the risk factors of heart disease, patient’s health promotion and lifestyle changes after CABG. Previous studies also have measured cardiac rehabilitation among cardiac patients. They have mainly focused on outpatient rehabilitation and cares and a few have long-term follow-up except for at discharge of the program (Clark, A. M., Hartling, L., Vandermeer, B., & McAlister, 2005; Tzou, Vitcenda, & McBride, 2004). The aim of present study was to clarify effect of health promotion programs and education on patients that hospitalized in Shiraz Major Hospitals after CABG and follow-up during three months.

## 2. Methods and Materials

This quasi-experimental research was designed to assess the effect of HPPs on patients’ lifestyle after CABG hospitalized in the years 2011-2012 in Shiraz Hospitals.

The research sample was 80 patients (with a mean age of 45-55 years) who had been hospitalized in cardiac surgery wards, participated in this study. Including criteria were all patients after CABG that inpatient in hospitals in Namazi, Faghihi and MRI hospitals. Patients with diabetes, substance abuse, age over 65 years and underlying disease were excluded of study.

All the selected patients were divided into two experimental (40) and control (40) groups through simple randomized method. Both groups were assessed and evaluated at different phases (pretest & post test) on different aspects of lifestyle.

### 2.1 Instrument

Baseline characteristics: age, gender, risk factors such as hyperlipidemia, physical activity time and diet were collected.

The instrument applied to measure the health promoting lifestyle was based on the (HPLP) questionnaire were purchased and with permission of Walker, Sechrist and Pender (1987) who designed this questionnaire. Then it validated and determined with Chronbach’s alpha 83% in IRAN (Safabakhsh & Moattari, 2005). The Health promotion lifestyle profile II (52 items) which measured the following aspects of lifestyle: Health Responsibility (9 items), Physical activity (8 items), Nutrition (9 items), Interrelationship (9 items), Stress Management (8 items) and Spiritual Growth (9 items) which are scored on a Likert scale ranging from 1 to 4 (4 for always and 1 for never). This questionnaire completed by both groups (experimental & Control) in two times: the first on ward during discharge and second time after three months.

### 2.2 Ethical Considerations

Ethics committee of Shiraz University of Medical Sciences approved this study.

The study was performed by considering moral aspects like conscious willingness of participants to keep their information and identity secure.

### 2.3 Intervention Program

The aim of the educational intervention period was to obtain positive changes of behavior and life style modifications in the six aspects of the HPLP in experimental group.

To assess the experimental group, the risk factors for learning needs were detected using assessment form 5A (Assess, Advice, Agree, Assist, and Arrange). This assessment form has already applied by American Cancer Instructions (http://www.cancer.org/search/index?QueryText=fiveAassessment). Based on individual assessment, each patient received the instructional program and was consulted on diet (low salt, low fat and the family’s roles in diet), physical activity (walking program during the one-week and increase rates and duration of walking and the family roles in physical activity), stress management (teaching how to reduce stress and the family roles in stress management) in three times: on first day before discharge, discharge day and at the first week after discharge. In all sessions which they lasted 30 to 45 minutes, one of the family members should be present. An instructional package containing necessary information related to lifestyle modification (about diet, stress management and physical activity) as well as a tape record of relaxation technique was given to the patients. The training program was followed twice during three months (on 6th & 10th weeks) by phone call after discharge of hospital. Patients and their family were reminded about diet, exercise and managing stress during this period.

The post-test period began three months after the completion of HPP. As in the pre-test period, the HPLP was administered to experimental group to evaluate changes in the overall score as well as in behaviors related to the six subscales. After three months, all patients were invited to the hospital and they were politely asked again to complete the questionnaire. After completing the questionnaires, training package and tapes containing relaxation training program was given to patients in the control group. The obtained data were collected before and after training process.

### 2.4 Data Analysis

Data were manually entered into SPSS version 21(IBM Corp, USA) by one the authors and analyzed. The within group differences between before and three months after intervention were analyzed with a paired student t-test.

The between group differences in change between before and three months after intervention were analyzed with a student t-test. A P value <0.05 was considered as significant.

## 3. Findings

Mean age of patients were 35-45 year (11.5%), 55% in range 35-45 and 33. % had more than 45. Participants were (67.5%) male and 26 (32.5%) were female. Risk factors were prevalent in these patients: smoker (28.2%); more than moderate overweigh (23.8%); diabetics (18.8%); fatty diet (76.3%); family history of CAD (22.5%); Hyperlipidemia (40%); and sedentary lifestyle (76%). Score of stress management (p=.036), diet (p=.002), Spiritual Growth (p=.001) and interrelationship (p=.002) increased in experimental group after intervention [Table 1]. Mean scores after three months in the control group had no significant changes; except responsibility for health, Interrelationship and Stress management (p<.05) [Table 2]. Results of the study revealed that comparison the scores of the experimental group were significantly different from the control group in all lifestyle aspects except for spiritual growth (p=0.1). There are significant differences in the Stress Management (p<.05), Diet (p<.05), Health Responsibility (p<.05), Physical activity (p<.05) and Interrelationship (p<.05). [Table 3]

**Table 1:** Lifestyle aspects, in study participation: experimental pre & post intervention (mean SD)

Lifestyle aspects	Pre intervention		Post intervention		T	P
	
	mean	SD	mean	SD		
Health Responsibility	18.2	.213	3	.24	4.1	.008
Nutrition	19.7	.135	31.3	.258	.62	.001
Physical activity	11	.135	22	.442	.30	.058
Interrelationship	25.6	.32	31.5	.193	.48	.002
Stress management	22.5	.308	31.5	.168	.33	.036
Spiritual growth	31.6	.26	32.2	.25	.49	.001

**Table 2:** Lifestyle aspects in study participation: control group, primary and after 3 month (Mean SD)

Lifestyle aspects	Primary		After 3 month		T	P
	
	mean	SD	mean	SD		
Health Responsibility	17.8	.25	18.3	.34	2.4	.001
Nutrition	19	.24	18	.28	1.8	.265
Physical activity	10.5	.99	10.3	.98	1.4	.098
Interrelationship	23.1	.35	22	.35	1.3	.004
Stress management	20.6	.32	18.7	.34	3	.046
**Spiritual growth**	**29**	**.22**	**28.5**	**.34**	**7.2**	**.706**

**Table 3:** Lifestyle aspects in study participation: experimental & control groups (Mean different SD)

Lifestyle aspects	Experimental		control		T	P
	
	Mean diff	SD	Mean diff	SD		
Health Responsibility	1.81	.251	.05	.288	7.29	.001
Nutrition	1.61	.221	-.1	340.	1.33	.001
Physical activity	1.1	.421	-.02	.118	2.17	.001
interrelationship	.59	.285	-.09	.369	9.12	.001
Stress management	.9	.298	-1.9	.387	1.9	.001
Spiritual growth	.06	.258	.5	.421	1.5	>.120

About self-actualization, results for experimental group showed higher mean scores due to the various types of activities in which they participated, e.g. social assistance. In health responsibility, the mean scores of the experimental gropu were initially higher and remained so throughout the follow-up. In physical exercise, the lower mean scores of the control group compared with the experimental group were probably due to their more sedentary lifestyle. To the fact that, in spite of their knowledge about health cares, they had not known its real importance. The experimental group started with higher scores in physical exercise because prior to participation in the study, they have already been in a system of programmed physical activity two to three times a week.

In the nutrition-related aspects, the median scores showed that the experimental group has had a healthier diet. As for interpersonal support, greater change in behavior was a result of the intervention. This may be due to the support they found in their community and the emotional relationships developed there. In stress management, the median scores showed that management stress in experimental group was better than the control group.

## 4. Discussion

This research has conducted to determine the impact of a Health Promotion Program (HPP) on behavior in terms of the dimensions of the Health Promoting Lifestyle Profile (HPLP) in patients after Coronary Artery Bypass Graft (CABG. The results showed that the prevalent risk factors were smoker (28.2%); more than moderate overweigh (23.8%); diabetics (18.8%); fatty diet (76.3%); family history of CAD (22.5%); Hyperlipidemia (40%);. This findings are in line with Kiani, Hessabi and Arbabisarjou (2015) finding. They concluded that 17% of patients (coronary artery diseases history), 25.5% (hypertension history), 26% (diabetes history), 15.5% (cholesterol history), 13% (smoking) and 3% have reported CABG history.

The results of this study cleared that HPPs were effective on life style changes and health promotion in patients after CABG. During three months follow-up after CABG has reduced the risk factors for CAD. After three months follow-up, the major influence was on lifestyle change such as diet, physical activity and weight control. These findings were in line with previously reported results of Dantas et al. (2002) that were significant differences in the Stress Management, Diet, Health Responsibility, Physical activity and Inter relationship in patients after CABG (Dantas, Aguillar, & dos Santos Barbeira, 2002). Carreno et al., (2006) has conducted a study with health promotion programs in Adventist and non-Adventist women. It has found significant differences in the stress management, diet, health responsibility, physical activity and interrelationship in experimental group. Muto et al., (2003) have shown that a HP programs developed for sales representatives to improve cardiovascular risk factors was effective in reduce of total cholesterol level. The main programs consisting of health education for thirty minutes by community health nurses (CHNs), and the follow-up programs were consisting of telephone counseling by CHNs (Muto, Hashimoto, Haruyama, & Fukuda, 2006). The same investigators reported by Chun-Gill Kima (2006) after a follow up did difference in life style aspects. The completion of a nurse-managed health-promotion program by institutionalized elderly women resulted in significant positive changes in the total scores of cardiovascular risk factors, the performance of health behaviors and, life satisfaction (Kim, June, & Song, 2003). The findings of the present study suggest that nurses can play an important role in improving patient’s health. Telephone follow up provided an opportunity to identify and overcome barriers to change of life style. Through telephone contacts after discharge, nurses have the opportunity to help patients develop strategy for change lifestyle (Koch et al., 2007). A prevention or health promoting programs with a focus on physical activity and diet counseling followed by structured meeting can favor-ably influence several risk factors for cardiovascular disease and quality of life in subjects. These finding have important clinical implications for CABG patients. Patients who had positively altered health behaviors, maintained the change only in the short-time 2 (Clark et al., 2005).

Thus future research should focus on the long term health lifestyle. There is also need to make patients chronic nature of cardiac disease and the benefits of maintaining a positive healthy lifestyle.

### 4.1 Clinical Implications and Suggestions

This study leaded to several clinical implications. HPP is a significantly under used intervention despite its demonstrated benefits to physical and psychosocial outcomes after CABG. The findings of the present study suggest that nurses can play an important role in improving patient attendance at HPP and thus may improve patient outcomes after CABG. Strength points of this study are present of patient’s family members during education and their participation in lifestyle change programs. Also we follow up this programs thought family and follow up for 3 months. Many patients though that post CABG surgery does not require modify diet. We started these programs when patients were in hospital. The results of this study can be used for the health professionals, particularly nurses who are working in heart surgery wards. It is suggested that further research follow-up the long-term lifestyle change programs for six months and one year after CABG.

## 5. Conclusion

Overall, the results showed that health promotion programs after CABG can reduce the risk factors of the disease in patients. Evidence suggested it is better to begin education programs while the patient is in hospital still (Weberg et al., 2013).

The presence of family members and follow up programs by health care providers can help sustain these programs. Patient education is essential at the time of discharge but not enough. These programs should be pursued or refer patients to clinics and rehabilitation centers.
